# Recent Developments on Sequence Spaces and Compact Operators with Applications

**DOI:** 10.1155/2015/410652

**Published:** 2015-01-29

**Authors:** S. A. Mohiuddine, M. Mursaleen, Józef Banaś, Suthep Suantai, Abdullah Alotaibi

**Affiliations:** ^1^Department of Mathematics, Faculty of Science, King Abdulaziz University, P.O. Box 80203, Jeddah 21589, Saudi Arabia; ^2^Department of Mathematics, Aligarh Muslim University, Aligarh 202 002, India; ^3^Department of Mathematics, Rzeszów University of Technology, Aleja Powstańców Warszawy 8, 35-959 Rzeszów, Poland; ^4^Department of Mathematics, Chiang Mai University, Chiang Mai 50200, Thailand

The aim of this special issue is to focus on recent developments and achievements in the theory of function spaces, sequences spaces and their geometry, and compact operators and their applications in various fields of applied mathematics, engineering, and other sciences. The theory of sequence spaces is powerful tool for obtaining positive results concerning Schauder basis and plays a fundamental role in creating the basis of several investigations conducted in nonlinear analysis. The compactness is very often used in fixed point theory and its applications to the theories of differential, functional differential, integral, and integrodifferential equations.

The research papers in this special issue cover various topics like geometry of Banach spaces, Schauder basis and dual spaces, sequence spaces and their topological and geometric properties, approximation of positive linear operators by matrix and nonmatrix methods, convergence and stability results for iterative process, applications of iterative methods to a nonlinear integral equation, Littlewood-Paley operators on Morrey spaces, matrix transformations between certain sequence spaces, measures of noncompactness and their applications in characterizing compact matrix operators, fixed point theorems and their applications, and so on.

